# Robustness during Aging—Molecular Biological and Physiological Aspects

**DOI:** 10.3390/cells9081862

**Published:** 2020-08-08

**Authors:** Emanuel Barth, Patricia Sieber, Heiko Stark, Stefan Schuster

**Affiliations:** 1 RNA Bioinformatics/High Throughput Analysis, Faculty of Mathematics and Computer Science, Friedrich Schiller University Jena, 07743 Jena, Germany; emanuel.barth@uni-jena.de; 2 Matthias Schleiden Institute, Bioinformatics, Friedrich Schiller University Jena, 07743 Jena, Germany; patricia.sieber@uni-jena.de; 3 Institute of Zoology and Evolutionary Research with Phyletic Museum, Friedrich Schiller University Jena, 07743 Jena, Germany; heiko@starkrats.de

**Keywords:** age-related diseases, failure accumulation, healthy aging, immunosenescence, robustness, theories of aging

## Abstract

Understanding the process of aging is still an important challenge to enable healthy aging and to prevent age-related diseases. Most studies in age research investigate the decline in organ functionality and gene activity with age. The focus on decline can even be considered a paradigm in that field. However, there are certain aspects that remain surprisingly stable and keep the organism robust. Here, we present and discuss various properties of robust behavior during human and animal aging, including physiological and molecular biological features, such as the hematocrit, body temperature, immunity against infectious diseases and others. We examine, in the context of robustness, the different theories of how aging occurs. We regard the role of aging in the light of evolution.

## 1. Introduction

Aging is a very complex process that can be defined, amongst others, as an accumulation of protein damage [1,2,3,4,5]. The mechanisms and consequences of aging are still not completely understood, and many questions remain open. Due to increasing life spans and a higher relative number of elderly people, it becomes more and more important to understand the underlying mechanisms to ensure healthy aging [6]. Analyzing the causes of aging is of interest in biology in general, for example, for understanding why some animal species are long-lived or short-lived.

Several hundred theories of how and why aging is happening have been suggested within the last decades [7,8]. In general, the manifold theories about the source of aging can be divided into two groups: the programmed aging theories and the failure accumulation theories [8]. Whereas theories of the first group argue that aging is a purposeful and, to some extent, inherently programmed or guided process, theories of the latter group have a contradicting view on aging. They focus on the organism’s inability to maintain its vital functions in the long term due to a multitude of different internal and external stochastic sources of damage [9].

As we use the term robustness throughout this review, the definition of that term is worth being recapitulated. In the theory of dynamical systems, a distinction is made between robustness and stability. While stability refers to the capacity of a system to go back to the original state after perturbations of variables (e.g., metabolite concentrations), robustness refers to the capacity to be (more or less) insusceptible to perturbations of parameters (e.g., temperature) and is often used synonymously with homeostasis [10]. Robustness means that a system can maintain functionality over a wide range of conditions [11]. It is an essential feature of evolutionary systems providing adaptive and protective mechanisms to cope with intrinsic and environmental challenges [12]. Note that the words ‘conditions’ and ‘challenges’ can be understood in the sense of ‘parameters’ and ‘parameter changes’.

Robustness can have a positive impact on the fitness and survival of an individual, unless the costs involved exceeding the benefit. In addition, the stable situation due to robustness is not limited to the current state but may change into another stable situation. This means that it can change over time but can still be robust within certain limits [11].

In the traditional view, the aging process is mostly associated with a decline in functionality and an accumulation of damage [1,2,4,13,14]. The focus is often on the course of deterioration in function, as expressed, for example, by the term “senescence“ (e.g., immunosenescence). This view is currently corroborated by the COVID-19 pandemic, which is particularly, but of course not exclusively, dangerous for the elderly [15,16]. However, many features of living organisms are surprisingly robust during the lifetime [11]. Examples are provided by the hematocrit, the immune defense against infectious diseases, and body temperature. These and other examples will be discussed in this article. They show that the decline with increasing age is not as drastic and fast as expected.

**Figure 1:**
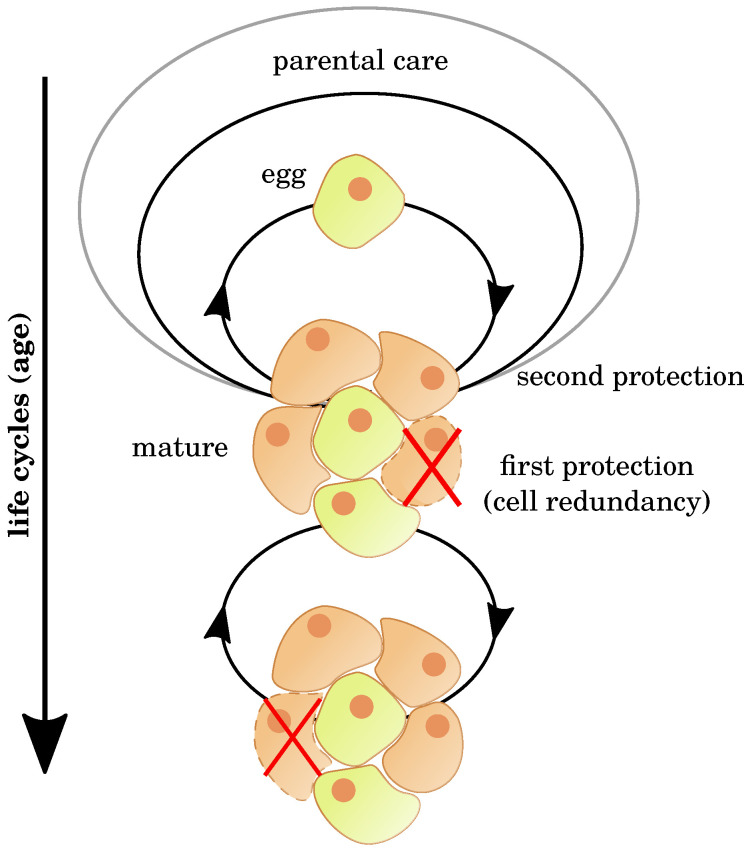
Cell lines can protect themselves against damage. With ongoing life cycles, cells divide and progress intro mature cells. That process differs in the speed and number of cells depending on the tissue type. Cells in multicellular organisms can be protected by neighbouring cells (orange) and other individuals (parental care and broader). Damage, however, is not avoidable and causes the death of single cells (crossed out), but the cell line itself survives and stays robust. In theory, all germ cells could live endlessly.

Importantly, we observe immortality in the case of unicellular organisms such as bacteria and archaea. In principle, they can divide forever (see Figure 1). Some of them, such as *Caulobacter crescentus*, do so in an asymmetric way, so that one daughter cell receives damaged proteins, while other bacteria divide symmetrically [11,17]. Aging in unicellular organisms is a controversial topic. Some studies did not find aging in symmetrically dividing unicellular species, while others did [17]. Below, we will not discuss unicellular organisms because this is beyond the scope of the present review. We have mentioned them here only to stress that immortality is realizable in principle.

Furthermore, many plants such as the creeping buttercup (*Ranunculus repens*) or St. Bernard’s lily (*Chlorophytum comosum*) are practically immortal when reproducing vegetatively [18], and so are the cells in the germline of sexually reproducing multicellular organisms and in immortal cell lines used in scientific research, such as the HeLa cells (see Figure 1) [19].

The wide-spread notion that aging implies impairment may result from a misinterpretation of the second law of thermodynamics. That law states that entropy, which is usually interpreted as a measure of disorder, can only increase in a closed system or remain constant in complete thermodynamic equilibrium. This putative increase in disorder would imply a decline in function. However, living organisms are open systems. By the uptake of free energy in the food, the increase in entropy can be prevented. Thus, thermodynamics does not immediately prevent immortality.

A point that is harder to rebut is that all living organisms have to cope with damage, e.g., by radiation. Therefore, they involve repair mechanisms, for example, for correcting mismatches in the DNA double-strand or reverting the oxidation of methionine. The problem is that the repair mechanisms can also be damaged, requiring a second-order repair system and so on. As an infinite series of such systems is impossible, aging seems to be unavoidable. The problem of infinite regress had been analyzed by Robert Rosen [20]. He suggested that living organisms could solve the problem in that they are closed with respect to efficient causation, in the sense of Aristotle’s causa efficiens. The philosopher Aristotle distinguished four causes: material, efficient, formal and final causes. The efficient cause consists of things apart from the thing being changed, which interact to bring about the change [21]. Thus, the efficient cause of the repair of a repair mechanism could consist of a second-order repair kit. If a repair mechanism can repair itself or two or more such units form a closed cycle, the system is closed with respect to efficient causation [22]. While Rosen did work on senescence [9], he did not specifically apply the idea of closed efficient causation to aging and senescence. It is still an open question whether repair mechanisms can form a cycle.

In the context of evolution, individuals are not “essential” for the population after their reproduction phase (except for social communities, e.g., presociality or eusociality). One could expect a faster decline after the reproduction phase, as is the case for some species, for example, Labord’s chameleon (Hamadryas baboon) and the fruit fly. However, this is not the case for humans, and there are further species with high longevity with an early reproduction (in relation to the life span), such as the Bowhead whale, Red sea urchin, and Galapagos tortoise [23]. In this case, robustness is a factor of evolutionary behavior [11].

In this review, we first summarize the different theories of aging (Section 2) and then take a fresh look at various aspects of robustness during aging on different biological levels, including physiological and molecular biology aspects, and referring to examples from various animal species (Section 3 and Section 4). Finally, Section 5 provides a summarizing discussion of the robustness in aging processes.

## 2. The Different Theories of Aging

Due to its incompletely understood nature, there is a decades-old and still ongoing debate about the true source of aging, giving rise to a variety of competing theories. There is no doubt in the scientific community that not all facets of aging can be explained by just one or a few single genes or gene regulatory pathways. However, the molecular origin, or in plain words the “starting point” of aging, is still intensely discussed. Here we give an overview of recent but differing arguments on how aging happens on a molecular level. Since there are several hundreds of partially overlapping aging theories [7], we will focus on the most discussed ones, providing a basis of what currently are believed to be the potential driving factors of aging [24,25,26]. These driving factors will be in part in contrast to the robustness factors that we will discuss in the remaining sections.

**Figure 2:**
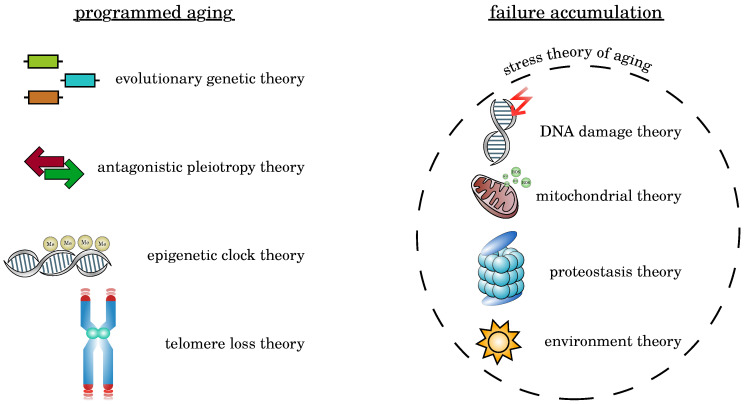
Overview of the presented aging theories. The different theories regarding the main driving factors of aging can be divided into two groups: programmed aging theories, stating that aging results from a genetic program (see Section 2.1, Section 2.2, Section 2.3, Section 2.4 and Section 2.5) and failure accumulation theories, stating that aging results from unavoidable, accumulating damage (see Section 2.6, Section 2.7, Section 2.8, Section 2.9 and Section 2.10). The collection of aging theories presented here is far from complete, but it summarises the current theories.

In general, each theory about the molecular source of aging belongs to one of two classes: the programmed aging theories and the failure accumulation theories [8] (see Figure 2). In the following four subsections, we will review selected programmed aging theories, discuss them in the subsequent subsection, and review five failure accumulation theories. The selection of theories to be discussed is made in view of the topic of this review and is, to some extent, unavoidably subjective.

### 2.1. Evolutionary Genetic Theory

This theory is one of the oldest regarding the reason and purpose of aging and argues that aging is built into the system by specific genes so that older individuals die and younger ones take over [27]. Therefore, there has to be an evolutionary robust genetic program that allows such a “turnover of generations”. This idea was pushed by the discovery that in several species, the knockout or inactivation of certain genes can prolong the life-span of individuals [28,29,30,31,32]. Consequently, their activation was correlated with the decline of cellular functions, and some evolutionary biologists argued that the existence of those genes would not make sense concerning natural selection, if not for triggering death [33,34,35].

### 2.2. Telomere Theory

Telomeres are the terminal regions at the ends of each chromosome arm in eukaryotes and consist of many relatively short nucleotide repeats [36]. They protect the genetic information because with every genome duplication during a cell’s division the chromosomes get shortened at their terminal regions due to the end replication problem [37]. Therefore, with every cell division, the telomeric regions get shorter (from around 11 kb in human newborns to less than 4 kb on average in centenarians [38,39]), triggering the cellular senescence replication stop if a critical length is reached (also known as the “Hayflick limit” [19,40]). Cells in replicative senescence are unable to proliferate further, and their overall function is strongly diminished [41,42].

During an organism’s lifetime, most cells continuously undergo proliferation and cell division and reach the state of senescence at their own pace, which over time, leads to an accumulation of senescent cells accompanied by loss of function and integrity of the respective tissues. Some concluded from this observation that telomeres take the role of an internal “countdown” to keep somatic cells stable by restricting their division cycles, preventing them from turning into cancer cells but also regulate the maximum life-span of an individual [43,44,45].

### 2.3. Antagonistic Pleiotropy Theory

In 1953, Williams [46] argued that evolution would favor genes that have (small) positive effects during early life, even though they have negative effects during late life [47]. That is because an early advantage in replication outweighs harmful effects that occur later in a species’ life history, even at the price of aging and death. One of the most prominent examples is the senescence key gene *p53*, which suppresses cancer early in life, but is responsible for the accumulation of senescent cells at a later age [48]. One allele of the human complement factor H gene provides better protection against pathogenic bacteria and fungi but increases the risk to suffer later from age-related macula degeneration (AMD) [49]. This dilemma can be modeled by evolutionary game theory [50]. Since most wildlife animals die earlier than their life-span would allow, due to accidents, disease, starvation or predation, there has never been any evolutionary pressure on these pleiotropic effects [51].

### 2.4. Epigenetic Clock Theory

Epigenetic marks are defined as heritable and reversible chemical modifications of histone proteins in order to change chromatin structures (e.g., through lysine or cysteine methylation) or as direct changes of the DNA [52,53,54]. Especially the methylation of DNA (at cytosines) is known to be associated with the down-regulation of genes [55].

Many independent studies have shown that there is a strong correlation between aging and DNA methylation [56,57,58], leading to new hypotheses that there is an epigenetic program that restricts life-span by turning down genetic processes with time passing by, supporting the ideas of a robust turnover program of the evolutionary genetic theories (see Section 2.1) [59,60]. This process was observed for many different tissues during aging, leading to the identification of specific tissue-dependent and tissue-independent DNA methylation sites [61]. These sites could also be used to predict the biological age of specific tissues, establishing the concept of the “epigenetic clock” [53,62]. However, this theory still competes with the stochastically occurring epigenetic changes with age (also known as “epigenetic drift”), arising from random (de)methylation processes and displaying a large variability across individuals [63,64,65,66].

### 2.5. Discussion of Programmed Aging Theories

All four theories implicitly suggest that the aging process is directly modulated by evolution for every species and that it could be explained by natural selection. This is also the principle of the disposable soma theory, which describes aging as an evolutionary trade-off between senescence and growth [67]. In a sense, these theories are in better agreement than the failure accumulation theories with the view that many features show robustness during aging because the former theories assume that failure accumulation is not an issue.

However, there are strong arguments against such evolutionary implications. For example, different studies on the mortality of wild animals showed that in many species almost all individuals die relatively early due to predation, cold or starvation, and only a few reach an age were factors like the accumulation of senescence, or epigenetic methylation marks become relevant (for a brief review, see [68]). This observation implies that there is little or no selective pressure on potential genetic aging programs. Consequently, such genetic aging programs either do not exist or are evolutionary extremely robust. Especially the “epigenetic drift” could not be proved to be more than random changes that add up over time, eventually silencing critical cellular processes just by chance [64,69].

Most importantly, none of the theories can explain the non-existence of “cheaters”, i.e., individuals that benefit from a deactivated aging program, because the overall suggested evolutionary benefit applies only for the whole group of a species and not for an individual alone [8]. Nevertheless, there are still proven genetic pathways that, when modified, can shorten or prolong life in different animal models significantly but are mostly counted as longevity instead of aging processes.

In the following, we will introduce the main ideas behind the failure accumulation theories.

### 2.6. DNA Damage Theory

This theory is a generalization of the free radical theory of aging [70] and is based on the fact that cells can enter replicative senescence if their DNA is damaged beyond repair. Due to several internal and external sources of stress, different impairments of the genomic DNA accumulate, like DNA single or double-strand breaks, non-canonical mutations or chemical modifications of single bases.

If a cell cannot cope with the damage despite having various and complex DNA repair mechanisms senescence will be triggered to prevent the transformation into a cancer cell [14]. Within the state of senescence, a cell fully stops growing and replicating, however, not making room for a new completely functional cell, and thus preventing it from participating in sustaining tissue homeostasis. As a consequence, more and more cells enter senescence overtime, and organ functions decrease to a point where the maintenance of the whole body collapses.

### 2.7. Mitochondrial Theory

In principle, according to the same reasoning as in the DNA damage theory, the mitochondrial theory identifies the cell’s mitochondria as a source of harmful reagents for DNA, namely the reactive oxygen species (ROS) [71,72,73]. ROS are generated as a byproduct of the mitochondrial electron transport chain, the central energy-producing process within cells, and play a natural role in some cell signaling pathways [74].

Mitochondria hold their own DNA (mtDNA), encoding for integral proteins of the oxygen processing machinery. Since the mtDNA is close to the site of the metabolic production of ROS, it is incredibly susceptible to DNA damage induced by ROS overproduction. This can lead to a vicious cycle since somatic mutations in mtDNA result in impaired mitochondrial proteins and an increasingly dysfunctional electron transport chain, consequently producing even more harmful ROS [75]. As a consequence, mitochondria become less and less stable over time and slowly turn into internal genotoxic threats, culminating in the activation of replicative senescence or cellular death.

Nevertheless, there are conflicting experimental observations in different animals, showing an unaffected or even shortened life-span after a reduction of oxidative stress by mitochondria or the complete removal of mtDNA [76,77,78]. Furthermore, besides the mitochondrial decline during aging, the oxidative phosphorylation remains surprisingly stable (see Section 4.4).

### 2.8. Proteostasis Theory

Sometimes referred to as waste accumulation theory, it is based on the assumption that the main origin of internal cellular stress is caused by misfolded proteins and deleterious protein aggregations [79]. The argumentation is similar to the one above. The accumulation of disturbing proteins results, over time, in a self-enhancing cycle of stress-induced interference with molecular processes, which in turn leads to an increased formation of harmful proteins triggering senescence. Common examples of such events are neurodegenerative diseases like Alzheimer’s or Parkinson’s disease and cancer, which are known to be triggered by dysfunctional and constrained protein complexes [80].

Normally, certain proteins are responsible for the correct folding of the majority of newly translated proteins (so-called chaperones or heat shock proteins) or the adequate degradation of old and non-functional proteins (mainly the proteasomes). With age, these mechanisms become inaccurate and reduced, possibly due to different internal and external stresses, making cells incapable of controlling the natural protein turnover, and resulting in the above described fatal consequences [81]. One important phenomenon in this context is the non-enzymatic, spontaneous modification of amino acids within proteins, such as peroxidation, hydroxylation, and chlorination [5]. Interestingly, the splicing process seems to be stable with age and does not contribute substantially to the generation of deleterious proteins (see Section 4.3).

### 2.9. Environmental Theory

In contrast to the theories mentioned above, supporters of the environmental theory (or closely related theories) claim that cellular changes and damages causing aging are mainly driven by exposure to external stresses and outweigh all internal sources of cellular stress [55,82]. These environmental factors include ultraviolet light, diet, ingested or inhaled toxic chemicals, heavy metals, radiation, heat or cold exposure, and psychological stress. Besides the direct damage of DNA, proteins or entire cells, the main influence of these factors is reflected by the changes in epigenetic modifications (especially DNA methylation) [63,66].

The age-dependent epigenetic drift causes a general down-regulation of gene transcription, resulting in intracellular miscommunication and deregulation of important biological processes [83]. Different cohort and twin studies could prove a direct and strong connection between the rate of epigenetic changes and aging, so that the topic of epigenetics attracted much attention in the field of aging in recent years [84,85,86].

### 2.10. Stress Theory of Aging

This is currently the most widely accepted theory and explains that different causes for aging do not have to be mutually exclusive. Its key message is that aging is most likely a combination of the processes mentioned above, which act and interact in parallel at different functional levels [87,88,89]. The progression of aging is tightly linked to all kinds of stress that an individual experiences throughout its lifetime. The ability to deal with internal and external stresses by any means is used to determine the rate of aging [90]. This makes sense, of course, when one considers that many aging theories overlap in their argumentation and states that aging is most likely caused by the complex interplay of different molecular sources in which cellular stress appears to be one of the central points.

In the following sections, we will discuss various aspects of aging that, in contrast to most aging theories, do not decline but show some kind of robustness during aging.

## 3. Physiological Properties

### 3.1. Immune System

A favorite subject of age research is the immune system of humans and higher animals [91,92,93]. The term immunosenescence has been coined in this context, and the paradigm of decline during aging is quite dominant in this field.

However, it is worth noting that in the first few years of life, the fastest and most effective immune response is provided by the innate immune system. It cannot distinguish between desired and undesired immune responses and has no long-term memory [92]. In contrast, the adaptive immune system can generate antigen-specific immunity by precisely recognizing antigens, memory formation, and adaptive proliferation. This gives the body the ability to adapt to new pathogens and to become more robust throughout life.

In the first two decades of the life of a human, the diversity of the T cell receptor in the thymus creates the prerequisite for the recognition of foreign antigens [94,95]. They are then maintained by the homeostatic proliferation of naïve T cells. Through repeated exposure to pathogens throughout life, the adaptive immune system achieves better and better protection. Due to antigen exposure, more of the naïve T/B lymphocytes with a vast polyclonal repertoire of antigen receptors shift to memory T/B lymphocytes with only a limited antigen receptor repertoire [96]. This leads to a diminishing naïve lymphocyte pool in aged individuals [94] and makes them more susceptible to newly arising infectious diseases, such as West Nile fever or severe acute respiratory syndrome [97,98]. Furthermore, it causes an impairment of the response to vaccines, and requires additional use of adjuvants [99].

**Figure 3:**
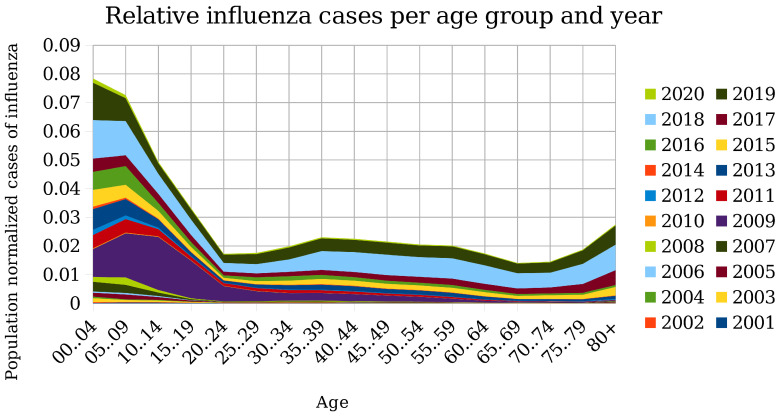
Number of relative influenza cases reported to the Robert Koch Institute, Germany (SurvStat@RKI 2.0, https://survstat.rki.de, Query date: 2020.02.17) divided by population size (United Nations, Department of Economic and Social Affairs, Population Division (2019). World Population Prospects 2019, Online Edition. Rev. 1) and grouped by age and year. Note the high incidence rate in children and youngsters. Moreover, there is a slight increase at the age above 75.

While in the case of COVID-19, the elderly show a drastically higher mortality rate ( [15,16]), it can be observed that older people have a better immune response to influenza viruses than young people (Figure 3) [100]. This observation depends on the statistics, though. Figure 3 shows the incidence rate rather than the mortality rate of infected individuals. As far as mortality is concerned, there are reports that pandemic spreads (H1N1 from 1918 to 1919, H2N2 from 1957 to 1958, H3N2 from 1968 to 1969, and H1N1 in 2009) mainly affect younger people and the subsequent epidemic spreads affect older people [101,102]. Another example challenging the paradigm of decline is that young cats are, in general, more susceptible to cat flu than adult ones (except very old ones) since the former have lost their maternally-derived antibodies without producing enough on their own already [103]. That disease is caused by feline herpesvirus type 1 (FHV) and feline calicivirus (FCV). Importantly, also, secondary infections pose a severe threat to kittens.

There is ample evidence that relevant biomarkers of the activity of the immune system indicate, on average, immunosenescence. However, when inspecting the results in more detail, it turns out that the variance of the data often increases during aging and that many older individuals tested show a perfectly active immunity.

For example, in Figure 5 in [2], the so-called CD4:8 ratio related to human T-cell phenotypes are plotted. While more and more individuals show an increased ratio in elderly people, many elderly individuals show a normal ratio. This is indicative of robustness. Another study revealed that the frequency and differentiation status of human γδ T-cells are only marginally influenced by age, except for people with a latent Cytomegalovirus infection [104]. No significant difference was observed in the net frequency of dendritic cells (DCs) producing IL-12 between young (23–34 years) and old (62–77 years) individuals [93]. In the same study, the total fraction of cytokine-producing cells (without subtracting background) did not differ between the two groups, and neither was the frequency of DCs producing IL-6 and TNF simultaneously significantly different between the young and old persons. However, the latter included many individuals with considerably fewer polyfunctional DCs than any of the young donors. Furthermore, the cytokine production level per cell was similar in young and old individuals [93]. In many immunological studies, the dependency on age is hard to discern due to large interindividual variability and a poor signal-to-noise ratio, which imply a significant variance of the data.

A striking feature in the immune system of the elderly is a chronically elevated low-level systemic inflammation [91,105]. This is partly due to the senescence-associated secretory phenotype (SASP) associated with senescent cells [106,107]. This includes secretion of higher levels of inflammatory cytokines such as IL-6, which have complex biological effects in ageing and cancer [108,109]. Thus, senescence can, in some instances, have opposite effects on tumorigenesis: in spite of its protective role, SASP can promote late-life cancer [106,109]. In general, chronic inflammation often correlates with carcinogenesis, diabetes, atherosclerosis, neurodegenerative diseases, etc. The exact causal relationship is not, however, known. It is unclear whether some other aging process independently leads to both disease and increased inflammation. The innate immune system increases the intensity and duration of the mediated inflammatory reactions, making older people susceptible to tissue-damaging immunity and inflammatory diseases [92,105].

### 3.2. Muscular System

From the viewpoint of robustness, it is interesting to consider the muscular system. It is well known that the mass, strength, and, thus, the performance of muscles decline in older individuals, for example, in humans [110] and the fish *Nothobranchius furzeri* [54]. For the population in most developed nations, cardiovascular disease is the most common cause of death [111]. Cardiac hypertrophy is a hallmark of cardiac aging [112]. High-performance sports cannot be practiced for very long in life. The potential of repair after muscle injury declines with age [110,113]. In the context of muscle aging, the mitochondrial theory (see Section 2.7), proteostasis theory (see Section 2.8) and others are worth being considered.

On the other hand, the muscular system is more robust than it may look at first sight. Some sportsmen show a remarkable performance even above 60 years of age, for example, in ironman triathlon [114]. The decline in muscle mass and function usually progresses at a low rate (primary sarcopenia). However, disease and injury usually accelerate this process (secondary sarcopenia) [110,115]. Secondary sarcopenia is influenced by immunological processes (e.g., increased IL-6 levels, see Section 3.1) [108,116]. In contrast, physical exercise has a positive effect on managing and preventing muscle loss [115]. Thus, primary sarcopenia is not only due to a direct effect by aging but also to an indirect effect by the sedentary lifestyle and lack of physical activity of many elderly people.

It has been found that the satellite cells within the skeletal muscle may have an essential role in aging-induced alterations. These constitute a pluripotent cell population with properties of stem cells in the adult skeletal muscle tissue. Some studies reported no differences in satellite cell counts in skeletal muscle between young and old men [117], while others did find a significant decline, associated explicitly with type II fibers, which contain less mitochondria [110].

A striking example of robustness is the heart muscle. The hearts of many animal species beat tirelessly until shortly before the end of life. The oldest non-colonial animal known to science is the ocean quahog *Arctica islandica* (Linnaeus, 1767) [118]. The known maximum life span of these bivalve mollusks is 508 years [119]. Within this enormous period of five centuries, the heart can beat more than 1.5 billion times [120]. Gerontological studies showed that *A. islandica* has increased resistance to several forms of stress compared to short-lived relatives [121,122]. Furthermore, Sosnowska et al. [120] could not find a significant difference of oxidatively damaged proteins in the heart muscle up to the age of 120 years. Successfully aging mammals, such as bats, also show lower protein oxidation (carbonylation) [123].

### 3.3. Vascular System and Body Temperature

An important medical parameter related to oxygen transport is the volume percentage of red blood cells, the hematocrit (Hct). The Hct value in healthy women (before menopause) amounts, on average, to approximately 40% and in healthy men to about 46% [124]. However, the values show considerable variations among individuals. Compared to that, there is no significant change depending on age, except for a slight increase in women after menopause. This is because menstruation causes a periodic outflow of erythrocytes, including defective ones. Since they are replaced by new erythrocytes, the percentage of defective erythrocytes is lower in women before menopause than in men [124,125]. Thus, the constancy in the Hct value is a further example of robustness during aging.

The average observed Hct value of about 40% in humans and many animals can be calculated based on optimality arguments [125]. If it were lower, fewer erythrocytes would be available to transport oxygen. If it were higher, the viscosity of the blood would be higher, so that it would flow more slowly. However, to maintain normal function, mammalian tissue oxygen concentrations must be tightly regulated within a narrow physiological range [126]. This explains, from a functional point of view, why it is beneficial to keep the Hct value constant during a lifetime. It is an interesting question of how the system that regulates the Hct value (which involves the hormone erythropoietin) achieves that robustness.

A crucial physiological parameter in homoiothermic animals is body temperature. That parameter is connected to the circadian rhythm so that it changes over the day. It is influenced by different internal and external factors, such as seasons, physical activity, nicotine and alcohol abuse, or menstrual rhythm [127,128]. The body temperature has been compared in human probands during normal aging and under the influence of age-related diseases. Some studies reported a slight decrease in mean body temperature, for example, a pioneering study by C.A. Wunderlich in the 1860s (see [128]). However, that appears to reflect nutritional, disease, and medication effects. Exclusion of these factors negates any relationship between age and mean body core temperature [127,129,130,131]. However, the variability of temperature increases in older people [130]. The tolerance to heat stress appears to be only minimally compromised by age, while the response to cold stress becomes weaker [130]. Various studies show that the body temperature has a comparable course of time over the day [129,130,131,132] with a minor decrease in amplitude in some individuals [128,130] (for a more detailed discussion of the circadian rhythm, see Section 4.1). Comparable results have been found in mice [133] and rats [134].

### 3.4. Brain Functionality

It is notable that making decisions or being attentive becomes more difficult with increasing age. Major intellectual achievements are often made by scientists in their twenties or thirties. On the other hand, there is a partially compensatory effect by growing life experience. Thus, many leadership positions in business, politics, and other areas are held by older people, and they are still capable of comprehending large systems or groups and making complex decisions.

Although the ability to handle task-related processes decreases and the prevalence of neurodegenerative diseases markedly increases with age, not all neuronal functions decline, and by far, not all elderly people are affected by these diseases [135,136,137,138]. For example, about 10% of individuals aged ≥65 years suffer from Alzheimer’s disease [138]. Campbell and colleagues conducted a human population-based study and observed that the auditory network and frontotemporal syntax network in the brain stay intact with increasing age [135]. Heard information can be processed by old persons in a comparable way as by young persons. The sensitivity of the corresponding networks does not change with age and shows that these cognitive abilities are robust [135]. Comparable results have been identified in population-based studies by Davis et al. [136] and Samu et al. [137], and show that language comprehension is not affected during healthy aging [136,137].

Furthermore, also the visuomotor processes stay partially robust [139,140]. Kadota and Gomi observed that the identification of visual stimuli and reaching them is not significantly different between young and old people [139]. This has been confirmed by Kimura and colleagues. They found out that the reaction towards object replacements stays high with age [140]. These examples show that neural functionality does not decline altogether.

### 3.5. Cell Redundancy

Most tissues consist of various cell types. For example, liver tissue includes hepatocytes, stellate cells, Kupffer cells, and others. Each cell type is present in numerous copies that have the same phenotypic structure and the same functionalities. In case of a malfunction or damage to an individual cell, this failure does not necessarily have an effect on the complete tissue. The functionality is preserved by other cells of the same type and, in some cases, by new differentiation of stem cells [141]. This behavior is analogous to the development of germ cells (see Figure 1) and shows how robust tissues are against damage.

The concept of redundancy has been investigated in skin wound healing in humans, where different growth factors are activated to ensure the healing process [142]. Redundancy plays a role not only at the tissue level but also in many cellular and regulatory mechanisms, such as the mechanisms of cell death [143] and transcription factor functionality [144] (see Section 4.2). Furthermore, immunological cascades have high redundancy with an important functional impact, including natural killer cells [145].

## 4. Regulatory and Molecular Aspects

### 4.1. Feedback Control

In the context of age research, Kriete discusses feedback loops as an example of mechanisms strengthening robustness [11]. Feedback loops ensure the functionality of certain processes as a control mechanism and prohibit a fast decline, such as in the NF-κB pathway. Feedback loops are often stable over time and show robustness also during aging in humans [11,146].

A further example is the circadian clock, for example, of body temperature and locomotor activity. That rhythm is based on at least two intertwined, conserved feedback loops [147]. In some individuals of laboratory animals and humans, a minor decrease in amplitude and a phase-advance can be observed, while many other individuals keep a normal circadian rhythm [127,128,129,130,131,133,134]. The results are heterogeneous with a sizeable inter-individual variance; even arrhythmia in some old rodents was observed [134]. The age-dependent changes of the temperature rhythm may partly be due to masking effects by the effect of locomotor activity on body temperature [127]. The rhythm can be kept robust with a stable concentration of SIRT1, a NAD${}^{+}$-dependent protein deacetylase, which introduces the loop and controls different regulators of the circadian clock [148,149,150,151]. The profiles of different rhythms seem to be altered differently, both in rodents and humans. Thus, their coupling seems to be reduced during aging. In elderly human subjects, some rhythms (e.g., temperature, melatonin) are often modified, whereas the time course of others (e.g., plasma cortisol, serum sodium concentration) remain unchanged [134]. Overall, the circadian rhythm is fairly robust into advanced age.

Theoretical studies in engineering science have shown that an increase in robustness always opens up a new side of fragility, however low the risk may be. For example, a negative feedback system such as a thermostat in a refrigerator usually improves homeostasis, but it can lead to dramatic system failure if it is damaged itself [152]. Such considerations have also been discussed in the context of aging [11]. An example discussed above is body temperature. While it provides advantages to keep it constant in homoiothermic animals, this is accompanied by a greater danger for life in the case of hypothermia. It is of interest to investigate the tight interrelation between robustness and fragility more systematically.

Furthermore hematopoiesis is subject to tight feedback control, in which the hormone erythropoietin (EPO) plays a vital role. For example, a progressively increased accumulation of EPO was observed during chronic hypoxia in young and aged mouse kidney [126]. However, under hypoxia, also the Hct value is often increased to compensate for the lower oxygen level. Interestingly, adaptation to hypoxia in high altitudes differs in different highlander populations. While indigenous people in the Andes mountains show higher Hct values than lowlanders, Tibetans have “normal” Hct values of about 40% and use other compensation mechanisms [153].

### 4.2. The Robustness of Networks

With increasing age of an organism, the effects of external stimuli accumulate. Tu and Chen [146] analyzed human expression data based on gene regulatory networks (GRNs) to analyze changes over time. They argue that robustness increases with regard to external stimuli (through a lower response to environmental stressors) and that more intrinsic variations are tolerated (for an explanation of the term robustness, see the Introduction.) The robustness of gene regulation might increase with increasing age [146]. Furthermore, GRNs contain various transcription factors (TFs). TFs ensure the functionality of cells and tissues and contain a certain redundancy, which increases its robustness [144]. Redundancy can also be observed in the feedback regulation of amino acid synthesis pathways, which is often implemented both by allosteric inhibitors and at the genetic level [154].

In the context of robustness, often, the so-called scale-free networks are discussed. These are networks in which a few nodes (so-called hubs) are densely connected to other nodes, while many nodes are only sparsely connected. The frequency of nodes as a function of connectivity follows a power law with a negative exponent [155]. It has been shown by data analysis that many biological systems such as biochemical networks [155] or protein-protein interaction networks [156] are scale-free. An important property of scale-free networks is to be quite robust to a random deletion of a node but fragile with respect to a targeted deletion of a hub. This implies that such networks per se cannot provide perfect robustness because perturbations may also hit hubs by chance.

### 4.3. Alternative Splicing—Functional Relevance vs. Noise

Splicing takes place regularly in eukaryotic cells to process pre-mRNA into mature mRNA. The splicing process is guided by the spliceosome, a macromolecular complex consisting of a high number of proteins and small RNAs [157,158,159]. The spliceosome ensures the (more or less) correct splicing process, including control mechanisms [157,160]. In addition, the complexity of the proteome can be widely increased by alternative splicing (AS), which leads to multiple isoforms of one and the same gene [159]. Missplicing of specific genes has already been linked to several age-associated diseases, such as progeria, Alzheimer’s disease, Parkinson’s disease, and cancer [161,162,163,164].

Alternative splicing produces different isoforms of proteins depending on gender, developmental stage, or environmental conditions. For example, coordinate alternative splicing changes drive embryo-to-adult transitions during postnatal heart development [165]. Moreover, age-related changes in isoforms have been found in many studies (for review, see [13]). For example, alternative splicing alterations have been found to be associated with mouse strain longevity [166]. Two questions arise in this context: to what extent and for what percentage of genes, the splicing process significantly changes, and whether the splicing process becomes more and more error-prone during aging, for example, by increasing malfunction of the spliceosome.

However, little is known on the global change between the most abundant isoforms of a gene in different tissues over time. A few studies identified individual isoforms derived from AS to be robust with age [167,168]. Lin et al. [168] discovered that the expression of one isoform of insulin-like growth factor-I (IGF-I), an important age regulator, is downregulated during aging in mice, while another one has a constant expression. A regulatory role for the various transcript isoforms of IGF-I has been suggested, while the main function appears to be the same for all. Only minor effects of aging on the distribution of isoforms could be found so far [169]. Aristorena and colleagues identified an increased expression of the low-abundant isoform of CD105 during aging in humans and mice, which introduces a decreasing proliferation [170]. Huan et al. point out that small proportions of AS change over time and are associated with age in different human tissues [171]. In a recent bioinformatics study, we found that AS is robust to aging for the majority of genes across various species, notably humans, mouse, zebrafish (*Danio rerio*), and killifish (*Nothobranchius furzeri*) as well as across different tissues [167]. This observation indicates that the whole splicing process seems to stay robust during aging. Furthermore, this is in line with the reasoning that the embryonic and adult phases are distinct developmental stages, while high age is not.

Regarding the spliceosome itself, mixed observations have been published on the expression change of genes coding for that macromolecular complex. Some studies have observed a stable expression of the main spliceosomal genes during aging with only a few significant changes for humans and mice, showing that the expression of essential components of the spliceosome seems not to change drastically during the age in these species [167,172].

However, other recent studies on various species and tissues observed an age-dependent decline in the expression of spliceosomal genes [166,172,173,174]. As a common assumption, it is argued that the age-dependent decrease in the spliceosomal activity leads to an inaccurate splicing process resulting in an accumulation of non-functional mRNAs. This additional “noise” in the transcriptome may reflect another source of stress to which aging cells are exposed and which contribute to the intrinsic obstacles to maintaining homeostasis.

These findings support the theory that the spliceosome and splicing products are changed but not deregulated globally, pointing to a relatively robust splicing process during aging. Changes in AS might even be a response to changing necessity. AS in human and rodent muscle cells during aging may be a means to react to damage (e.g., by ischemia or prolonged exercise) [169,175].

The degradation of incorrect (noisy) splice products may take place regularly as part of splicing control mechanisms [158,176,177]. Further studies suggest that a high proportion of AS is caused by stochastic slippage of the spliceosome [178,179,180]. Some of the isoforms without functionality are discarded, for example, by nonsense-mediated decay. Others, however, can be functional [178]. In the case of slippage, AS should be more or less invariant during aging because it is just a stochastic effect. An opposing view is that practically all AS events are physiologically advantageous [181], and noisy splicing might be an important evolutionary mechanism [180]. It might be used to regulate the overall expression of the corresponding gene in certain species [182,183]. This is an observation without taking aging effects into account. Thus, also aging data contain noisy AS events, which may be misinterpreted as being correlated with aging.

### 4.4. Energy Metabolism

Mitochondria are subject to damage due to ROS, and therefore, their function is more and more impaired during their lifetime (see Section 2.7). Thus, one would expect that the contribution of oxidative phosphorylation (cell respiration) declines during aging. Surprisingly, in many animals, the opposite is observed: its relative contribution (compared to glycolysis) increases [184,185]. Embryonic cells show a rather high fraction of glycolytic ATP production. This may be due to a lack of oxygen but is mainly due to the need for a high ATP production rate (rather than high ATP-over-glucose yield) in rapidly dividing cells. After birth, the fractional contribution of glycolysis permanently decreases. An interesting observation is that physical activity delays the decline in mitochondrial respiratory flux in aging mouse muscle [186].

The above observations can be interpreted in terms of robustness. Although the contributions of glycolysis and respiration are not constant, they develop in a way that opposes the effect of damage of mitochondria. Thus, that damage is over-compensated. This is only possible due to the remarkable robustness in mitochondrial energy metabolism.

Stadtman [187] suggested using the so-called energy charge, (ATP+0.5ADP)/(ATP+ADP+AMP), as a biochemical marker of aging. However, it was found that the intracellular ATP concentration does not change significantly during aging. For example, no difference in ATP or the total adenine nucleotide or creatine metabolite concentrations could be observed between three age groups of mice (1, 4, and 16 months) after recovery from cardioplegic arrest [188]. Thus, the energy charge and ATP concentration have not turned out to be reliable biomarkers of aging. Even quantities such as ROS production (e.g., superoxides), mitochondrial mass and mitochondrial membrane potential or markers of lipid peroxidation, which had been suggested as biomarkers, have not proven reliable and even been questioned [189].

## 5. Discussion and Conclusions

**Figure 4:**
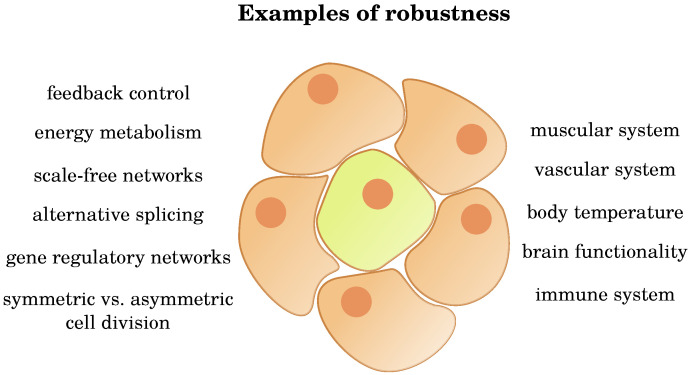
Examples of robustness discussed throughout this review. Some of the observed mechanisms apply to the cellular as well as the organ levels (**left**), others only to organ level (**right**).

In any scientific study, it is crucial to shed light on various aspects. While the effect of aging on the decline of physiological functions has been discussed in the literature at length and in much detail, the surprising robustness of many physiological functions has attracted much less attention (see Figure 4). In the above sections, we have outlined and discussed several examples of robustness on different organism levels, so that the paradigm of decline is worth being questioned.

It is worth mentioning that many studies on aging compare healthy with diseased or immune-deficient individuals and come to the conclusion that the effect of aging is especially severe in the latter [188,190]. This may, somewhat misleadingly, strengthen the focus on the negative aspects of aging. In the case of COVID-19, it appears that age plus comorbidity represent risk factors. Thus, it is hard to produce reliable statistics on the effect of age alone on susceptibility to that disease [15,16].

On comparing the programmed aging theories with the failure accumulation theories [8], it becomes clear that robustness is easier to explain by the former. If failures accumulate and cannot wholly be repaired, there is no chance to be perfectly robust. In contrast, if aging is programmed as a result of evolution, it can, at least in principle, be reprogrammed either by evolution or by human intervention to achieve immortality. For example, epigenetic modifications could be made differently.

A striking feature is that robustness during aging is often achieved by compensation mechanisms. For example, the decline in the efficiency of immune cells (immunosenescence) is partly compensated for by an increase in immune memory and perhaps also by a chronically elevated low-level systemic inflammation. It is, then, sometimes not easy to distinguish between harmful changes in aging and normal adaptation mechanisms. Moreover, robustness does not necessarily imply that every constituent remains constant; a variation in one constituent can be compensated by a variation in another, such as in the case of glycolysis and oxidative phosphorylation.

Moreover, it is interesting and useful to consider robustness during aging from the point of view of optimality. According to the concept of survival of the fittest, organisms should have optimal properties. In fact, optimality considerations are often used to compute parameter values on theoretical grounds [125,191]. Based on the plausible assumption that certain parameters, e.g., the Hct value [125], intracellular ATP concentration [191], and body temperature [130,192], have optimal values, evolution should have led to a constancy of that value, perhaps except for a decline just before death. The mentioned example parameters are really fairly constant, as discussed above. The process of aging leaves many open questions. It has been discovered that functionalities decline, balances and behaviors change [146,193].

Different studies with common scientific questions of aging do not always show unambiguous results towards the behavior over time. This demonstrates how difficult it is to understand the aging process thoroughly. Further, this is not the first article to point out that the understanding of aging cannot be easily described only by increasing damage [194,195]. Blagosklonny further argues that it is possible to repair germ cells and does not see a reason why this should not be possible for somatic cells as well. He states that aging may be a continuation of the cell and organism development, which makes it a robust process [195].

To keep age-related risk factors minimal, it is often suggested to have a healthy diet, stay physically active, and avoid specific stressors [196,197,198]. It is of particular importance that certain key factors of aging are known, such as SIRT1, mTOR, IGF-1, and AMPK [149,150,195]. Already with this knowledge, negative consequences of aging can be limited, certain function losses can be regained, and certain age-related diseases could be prevented [149,195,197]. The organisms’ functionality might be more robust with such an adapted behavior or treatment. This is an important point in showing the fragility in a close relationship to robustness during aging [149,198].

The above critical discussion of the paradigm of decline during aging is in line with recent research on the psychology of aging. Some authors in that field argue that aging can open up new opportunities such as less deadline pressure, more leisure time, financial independence, serenity, etc. [194,199]. Our general attitude towards aging may change to the positive, which may have implications for new working hypotheses also in biological age research.

## Abbreviations

The following abbreviations are used in this manuscript:

AS	alternative splicing
DCs	dendritic cells
ECM	extracellular matrix
Hct	hematocrit
mtDNA	mitochondrial DNA
ROS	reactive oxygen species
SPARC	secreted protein acidic and rich in cysteine
